# Oxygen and Hydrogen—A Correction

**Published:** 1891-11

**Authors:** E. V. Ross

**Affiliations:** Rochester, N. Y.


Editor of The Homœopathic Physician:

### OXYGEN AND HYDROGEN.—A CORRECTION.

Editor of The Homœopathic Physician:

In Vol. X of your journal, at page 400, under "Clinical Verifications," by Dr. Swan, he states that according to Hering's *Analytical Repertory of Symptoms of the Mind*, Oxygen has periodical symptoms every day *earlier*, and Hydrogen every day *later*.

On consulting the above-mentioned work, I read it just the reverse.

Respectfully,

E. V. Ross, M. D.

Rochester, N. Y., August 25th, 1891.

